# IMPACT OF EXERCISE/PHYSICAL ACTIVITY ON HEALTH SPAN: FROM MOLECULAR MECHANISMS TO FUNCTIONAL BENEFITS

**DOI:** 10.1093/geroni/igae098.0581

**Published:** 2024-12-31

**Authors:** Roger Fielding

**Affiliations:** Jean Mayer USDA Human Nutrition Research Center on Aging at Tufts University, Boston, Massachusetts, United States

## Abstract

Exercise/physical activity counters the onset and progression of chronic diseases (e.g., type 2 diabetes (T2D), cardiovascular disease (CVD), cognitive decline, and cancers), geriatric syndromes (e.g., frailty), and disability, even when introduced in later-life. Advancing chronological age is associated with well characterized declines in muscle size, performance, and physical functioning across multiple species. Underlying these age-related changes are declines in the force/power generating capacity of skeletal muscle that appear to be driven by changes in skeletal muscle contractile protein function, metabolic derangements, and alterations in neuromuscular activation. Age-associated changes in muscle gene transcription, epigenetic modifications, mitochondrial stability, and anabolic capacity, all suggest that fundamental changes in aging biology are responsible for these changes. Furthermore, many of these responses have been shown to be responsive to changes in regular physical activity suggesting a putative role for exercise/physical activity on health span with aging. In my presentation, I will review the underlying mechanisms of age-related changes in skeletal muscle mass and function and how changes in regular physical activity can attenuate or reverse the observed changes in mass and function that occur with advancing age with resultant improvements in health span.

